# Supplementary material for: Prognostic Implications of High-Degree Atrio-Ventricular Block in Patients with Acute Myocardial Infarction in the Contemporary Era
Source: J Clin Med. 2023 Jul 22;12(14):4834. doi: 10.3390/jcm12144834 (PMC10381467; doi:10.3390/jcm12144834)
Supplement: Supplementary file 1 [file jcm-12-04834-s001.zip › jcm-2465904-supplementary.pdf]

Journal of
Clinical Medicine

MDPI

Supplementary Materials:

Table S1. Comparison between our results with previous studies.

Authors	Incidence	Mean Age	Type of ACS	Initial TIMI flow = 0 *	Type of AVB	RCA involvement	Treatment
DIAMANTE (n=1109)	8.7%	64.0	STEMI	70.2%	HAVB	42.1%	PCI (93.1%) and fibrinolysis (6.9%)
Singh et al. [1] (n=59229)	2.9%	66.1	All	---	HAVB	35.7%	Angiography (62.1%): PCI (86.3%), fibrinolysis (12.9%) and CABG (5.1%)
Gómez-Talavera et al. [2] (n=913)	12.6%	63.2	STEMI	---	All	41.9%	PCI
Gang et al. [3] (n=2073)	3.2%	44%> 65 years	STEMI	---	HAVB	38.4%	PCI
Auffret et al. [4] (n=6662)	3.5%	62.0	STEMI	54.1%	HAVB	41.8%	Angiography (98.6%), PCI (74.0%), Fibrinolysis (12.4%)
Kosmidou et al. [5] (n=3115)	1.5%	59.9	STEMI	---	HAVB	41.6%	PCI
Aguiar Rosa et al. [6] (n=4799)	1.9%	63.7	All	---	Complete AVB	Inferior STEMI (23.41%)	In STEMI patients PCI (84.4%) and fibrinolysis (7.7%)
Kim KH et al. [7] (n=16536)	3.2%	66.2	STEMI	57.8%	HAVB	37.0%	PCI
Shacham et al. [8] (n=1244)	3.0%	62.0	STEMI	---	HAVB	---	PCI
Santos H et al. [9] (n=32157)	2.6%	71	All	---	HAVB	65.8%	PCI (85.7%), CABG (5.6%), hybrid strategy (2.6%)
Kawamura et al. [10] (n=1295)	6.3%	66	STEMI		Complete ABV	Non-anterior STEMI (RCA or LCX) 51%	PCI 100%

* in all patients. ACS = acute coronary syndromes; TIMI = thrombolysis in myocardial infarction; STEMI = ST-elevation myocardial infarction; AVB = atrioventricular block; HAVB = high-degree atrioventricular block; PCI = percutaneous coronary intervention; CABG = coronary artery bypass surgery.
